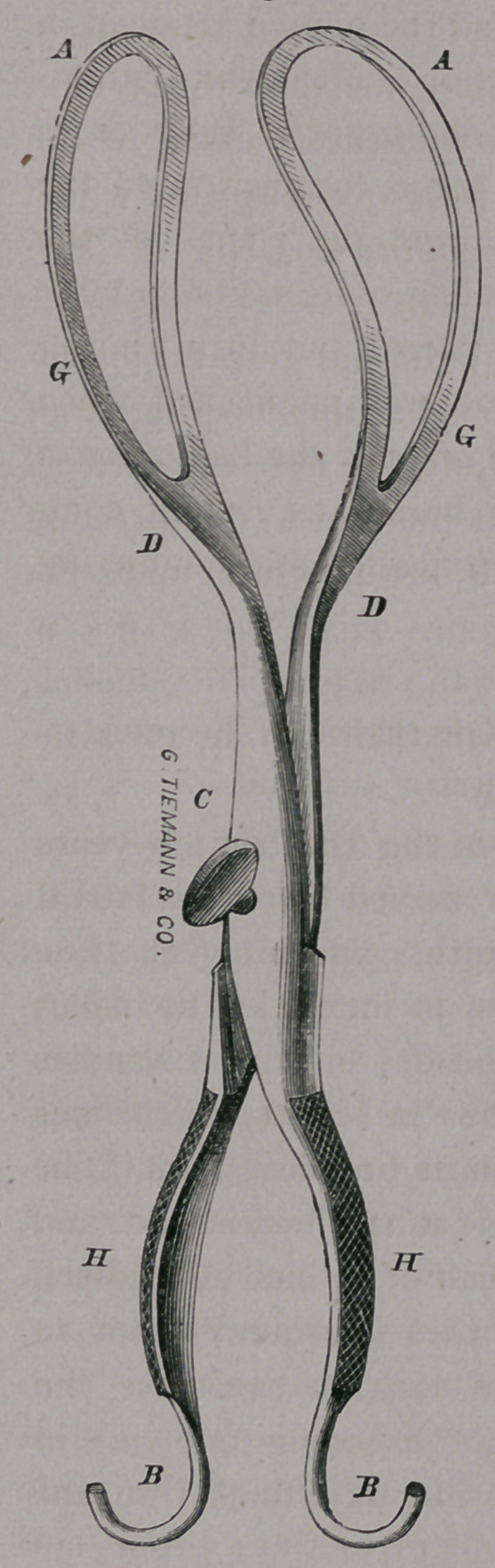# Extracts from a Paper on the Obstetrical Forceps

**Published:** 1881-11

**Authors:** James P. White


### Extracts from a Paper on the Obstetrical Forceps.

By Prof. James P. White, M. D., in Vol. XVII of this journal.

The instrument which I have used during the last few years is a long forceps, and is considerably curved upon its lateral aspects. It measures in its entire length, conforming the line measured to the curvature of the blades, 17 inches. The blades and their shafts to the pivot being about 9½ or 10, the handles about 7 inches. The blade is 6½ inches in length, and 7 lines at its narrowest point, and 2⅛ inches in its broadest point. The fenestrum is one and three-eighths (1⅜) at the widest part, and gradually diminishes to less than one-half of an inch at the heel. The inner or fenestral margin ot the blades is ground down so as not to exceed one-sixteenth of an inch in thickness, the width being scarcely 5½ lines, and not exeeding one line in thickness at its periphery, being considerably thicker in the center.

The shaft of the blade is scalloped out considerably toward the pivot, upon its inner surface, beyond the termination of the fenestrum, thus diminishing weight without lessening the strength.

The points of the blades when the instrument is closed are but 8 or 9 lines apart, and at the widest point they are 2 inches and 9 lines apart, on the upper or concave surface; whilst on the lower or convex surface, they are slightly more expanded. The shafts of the blades approach each other rapidly, but not abruptly.

The blades at the centre of the point deviate 3½ inches from the straight line in forming their second or pelvic curve. The entire thickness of the closed instrument at their point of junction is less than six lines. They are united by means of the German notch and button, or screw, which is countersunk in the female blade, and when the instrument is closed, the blades are held as firmly as by a pivot. The edges or shoulder of the mortise, or notch, are rounded, or pared off for four or five lines on either side, so as to incline the pivot to slide into the notch. The mortise is not carried very deeply towards the opposite side of the blade, which would greatly diminish its strength at this point.

The handles, unencumbered by the heavy wooden beams which are attached to the handles of many modern forceps, diverge in the centre to 1¾ inches, and each is expanded or flattened to 1⅛ of an inch in width at that point, and well roughened on the outer surface, so as to be securely grasped. Each handle is made concave on the inside and convex externally, thus diminishing its weight very much.

The points are contracted again, curved and polished, and will separately answer the purpose of blunt hooks. The one may be made to inclose a perforator, and the other a sharp hook or crochet. Each is made oval, and the sheath enveloping it is secured by means of a small transverse screw, which may be removed by the point of a pen-knife or scissors. The entire instrument is made of the German cast-steel, and is much better to be nickel-plated which prevents rust. It may be had of Tiemann & Co., No. 67 Chatham street, New York.

Here it is perceived we have a very light and graceful instrument of sufficient length to seize the head at the superior strait without difficulty, leaving the lock entirely free from the external organs. The curve is such, also, as to conform to the direction of the passage, without exerting injurious pressure upon the perineum. The shafts of the blade approximate so as not to distend the vulva before the descent of the head. They incline, however, so gradually as not to diminish their power, as is the case with the instrument of Dr. Hodge.

It will be found that the concavity of the fenestrum, beveling off the inner edges of the blades, will render it better adapted to fit accurately the parietal protuberances, and prevent those salient points from being injured or indented by the sharp angles usually found on the inner border of the fenestrum. Moreover, this is the widest part of the fœtal head and the surface to which the fenestrum is ordinarily applied, and if this margin of each blade be two or two-and-a-half lines in thickness, as is the case in many instruments, the amount of compression of the head must be three lines more in consequence of unnecessary thickness. One of the difficulties in application consists in uniting the blades. In the instrument represented this end is greatly facilitated, slightly lessening the weight at the same time, by cutting away the abrupt shoulders to the mortise, into which the screw easily glides, whenever it gets within these inclined planes. Again, whoever has been compelled to hold on to well polished round steel handles will readily appreciate the comfort, as well as sense of security, which a roughened and expanded surface must afford. The length of the handle may be increased and bent so as to form a blunt hook, and a very good perforator may be inserted into the extremity of one handle and a sharp hook into the other, which will answer very well if the work of destruction becomes unavoidable.

## Figures and Tables

**Figure f1:**